# Erroneous calibration of esophageal pressure in case of airway closure

**DOI:** 10.1186/s13054-025-05416-5

**Published:** 2025-05-02

**Authors:** Mattia Docci, Francois Beloncle, Arnaud Lesimple, Thomas Piraino, Davide Raimondi Cominesi, Andrea Restivo, Mayson L. A. Sousa, Emanuele Rezoagli, Alain Mercat, Jean-Christophe Richard, the CAVIAR Group, Laurent Brochard

**Affiliations:** 1 Keenan Centre for Biomedical Research, Li Ka Shing Knowledge Institute, Unity Health Toronto, Toronto, ON Canada; 2 Interdepartmental Division of Critical Care Medicine, University of Toronto, Toronto, ON Canada; 3 Medical ICU, University Hospital of Angers, Vent’Lab, University of Angers, Angers, France; 4 Med2Lab, Air Liquide Medical Systems, Antony, France; 5 Division of Critical Care, Department of Anesthesia, McMaster University, Hamilton, ON Canada; 6 School of Medicine and Surgery, University of Milano-Bicocca, Monza, Italy; 7 Department of Respiratory Therapy, Rady Faculty of Health Sciences, University of Manitoba, Winnipeg, Canada; 8 Department of Emergency and Intensive Care, Fondazione IRCCS San Gerardo dei Tintori, Monza, Italy

**Keywords:** Esophageal pressure, Calibration, Airway closure, Airway opening pressure, Positive pressure occlusion test, Baydur test, End-expiratory occlusion, End-inspiratory occlusion, Acute respiratory distress syndrome, ARDS

## Abstract

Airway closure results in a lack of communication between proximal and distal airways unless the airway pressure (Paw) overcomes the airway opening pressure (AOP). This has been described in patients undergoing mechanical ventilation with acute respiratory distress syndrome, obesity, hydrostatic pulmonary edema and during cardiopulmonary resuscitation. In these categories of patients, esophageal pressure (Pes) can guide the personalization of mechanical ventilation and calibration of the esophageal balloon is necessary to obtain reliable Pes measurements. The impact of airway closure has never been envisaged. This study investigated the impact of airway closure on the calibration of the esophageal balloon by the ∆Paw/∆Pes following a positive pressure occlusion test during passive mechanical ventilation. The calibration test was performed in twelve human cadavers with airway closure at end-expiration at different levels of positive end-expiratory pressure (PEEP) and at end-inspiration. The ∆Paw/∆Pes measured at end-expiration and at end-inspiration were significantly different when total PEEP was lower than AOP (estimated means 0.42 [0.40; 0.44] vs. 0.95 [0.92; 0.97], *P* < 0.001), while this difference was not observed when total PEEP was higher than AOP (estimated means 0.99 [0.92; 1.05] vs. 0.99 [0.92; 1.06], *P* = 0.854). These results were corroborated by observations during esophageal balloon calibration in two patients requiring Pes monitoring for clinical management. In case of airway closure, compression of the chest is not fully transmitted to the airways. This can lead to a conspicuous underestimation of the ∆Paw/∆Pes and poor reliability of this monitoring technique when the test takes place below AOP. Our results favor a positive pressure occlusion test performed during an end-inspiratory occlusion as the new standard of operative procedures for positioning and calibrating the esophageal balloon.

## Introduction

Airway closure results in a lack of communication between proximal and distal airways unless the pressure overcomes the airway opening pressure (AOP) [1]. This has been described in patients undergoing mechanical ventilation with acute respiratory distress syndrome (ARDS) [1], obesity [2], hydrostatic pulmonary edema [3] and during cardiopulmonary resuscitation [4, 5]. Esophageal pressure (Pes) serve as an advanced respiratory monitoring method with the potential to guide the management of mechanical ventilation. The accuracy of Pes measurement relies on positioning and calibration of the esophageal balloon [6]. When patients are spontaneously breathing, the Baydur occlusion test is the conventional test to validate the correct esophageal balloon positioning [7]. In passive patients, manual compression of the rib cage is applied during an expiratory pause, and simultaneous positive deflection of airway (∆Paw) and esophageal pressure (∆Pes) is compared (*positive pressure occlusion test*) [8–10]. In the absence of flow, when the airway is occluded, the pressure in the pleural space and Paw change equally in response to any forces acting on the chest wall. Therefore, a ∆Paw/∆Pes within 0.8–1.2 indicates that Pes measurement reliably approximates pleural pressure. Otherwise, the catheter needs to be repositioned and/or the balloon volume re-checked [7]. This is true only if the airways are open allowing transmission of alveolar pressure at the airway opening.

In a human cadaver model of cardiac arrest, our group showed that airway closure can limit the transmission of pressure to the airway during chest compressions for resuscitation [11]. We hypothesized that in case of airway closure, when a positive pressure occlusion test takes place below AOP, the resulting ∆Paw/∆Pes may be underestimated.

We aimed to investigate the impact of airway closure on the calibration of the esophageal balloon by a positive pressure occlusion test during passive mechanical ventilation and to show its application in two patients.

## Methods

### Human cadavers

We tested our hypothesis in a physiological cross-over study in human cadavers. The soft embalmed Thiel cadavers, which retain the body’s natural look and feel, reliably reproduce human respiratory mechanics while exhibiting a high prevalence of airway closure [11]. Considering the absence of cardiac artifacts, cadavers were particularly suited to study the Pes trace in a human model of airway closure. We studied twelve cadavers from a donation program at the Université du Québec à Trois-Rivières (UQTR, Québec, Canada). Each one was intubated, carefully suctioned, and equipped with a type of esophageal catheter (Nutrivent, Sidam, Italy), known to have a stable compliance of the balloon over a wide range of filling volumes, according to recommendations [6]. Before the beginning of the experiment, each cadaver was ventilated for 30 min in volume-control mode at 6 ml/kg of predicted body weight, positive end-expiratory pressure (PEEP) 5 cmH_2_O and respiratory rate 10 breaths/minute. Chest x-rays excluded pneumothorax and allowed confirmation of proper esophageal catheter placement. Each cadaver underwent a low-flow inflation (5 L/min) from 0 to 25 cmH_2_O to detect the presence of airway closure and measure the AOP. Baseline calibration of the esophageal balloon was performed above AOP. During the study protocol, a gentle positive pressure occlusion test was performed by manual chest compressions during end-expiratory and end-inspiratory occlusions. Each cadaver was tested at different levels of PEEP or at different inclinations of the trunk (0° and 30°) with or without a load on the chest (5-kg saline bag) to potentially modify the level of AOP. Paw, flow and Pes were recorded continuously using a dedicated monitoring device at a sampling frequency of 1000 Hz (BIOPAC, Systems Inc, USA).

### Statistics

Normality of data distribution was assessed using Shapiro–Wilk test. Data are reported as median (interquartile range). To consider the random effect of each cadaver in the different conditions, a linear mixed-effects model with repeated measures for each cadaver was used to assess whether performing the occlusion test at end-expiration vs. end-inspiration (fixed effects) influenced the value of ∆Paw/∆Pes. A random intercept model was fitted with the Restricted Maximum Likelihood method using the *mixedlm* package in Python 3.12.4 (Python Software Foundation, Delaware, USA). Analysis was performed for measurements overall and stratified according to total PEEP being below or above AOP. *P* value < 0.05 was considered significant. ΔPaw/ΔPes data pooled according to the occlusion test performed at end-expiration vs end-inspiration were expressed as estimated means [95% confidence interval] from the mixed models. LabChart-7-Pro (ADInstruments, Sidney, Australia) was used for off-line waveform analyses. GraphPad Prism 8.3.0. was used for preparing figures.

### Patients

We recorded waveforms in two exemplary patients from the Medical-ICU in University Hospital of Angers (France) and the Cardiac-ICU at Fondazione IRCCS San Gerardo dei Tintori (Monza, Italy), respectively. Both patients required Pes (Nutrivent, Sidam, Italy) for clinical management during passive mechanical ventilation. A positivepressure occlusion test to calibrate Pes was performed at end-expiration compared to end-inspiration (Patient #1) or at three different PEEP levels during an incremental PEEP trial (Patient #2). The presence of airway closure was assessed by a low-flow inflation (5 L/min). Paw, flow and Pes were recorded continuously at a sampling frequency of 25 Hz for patient #1 (Ohmeda software, GE Healthcare, Madison, USA), and 1000 Hz for patient #2 (PowerLab, ADInstruments, Sidney, Australia). Data from patients are included descriptively: no statistical test was carried out.

## Results

### Human cadavers

**Fig. 1:**
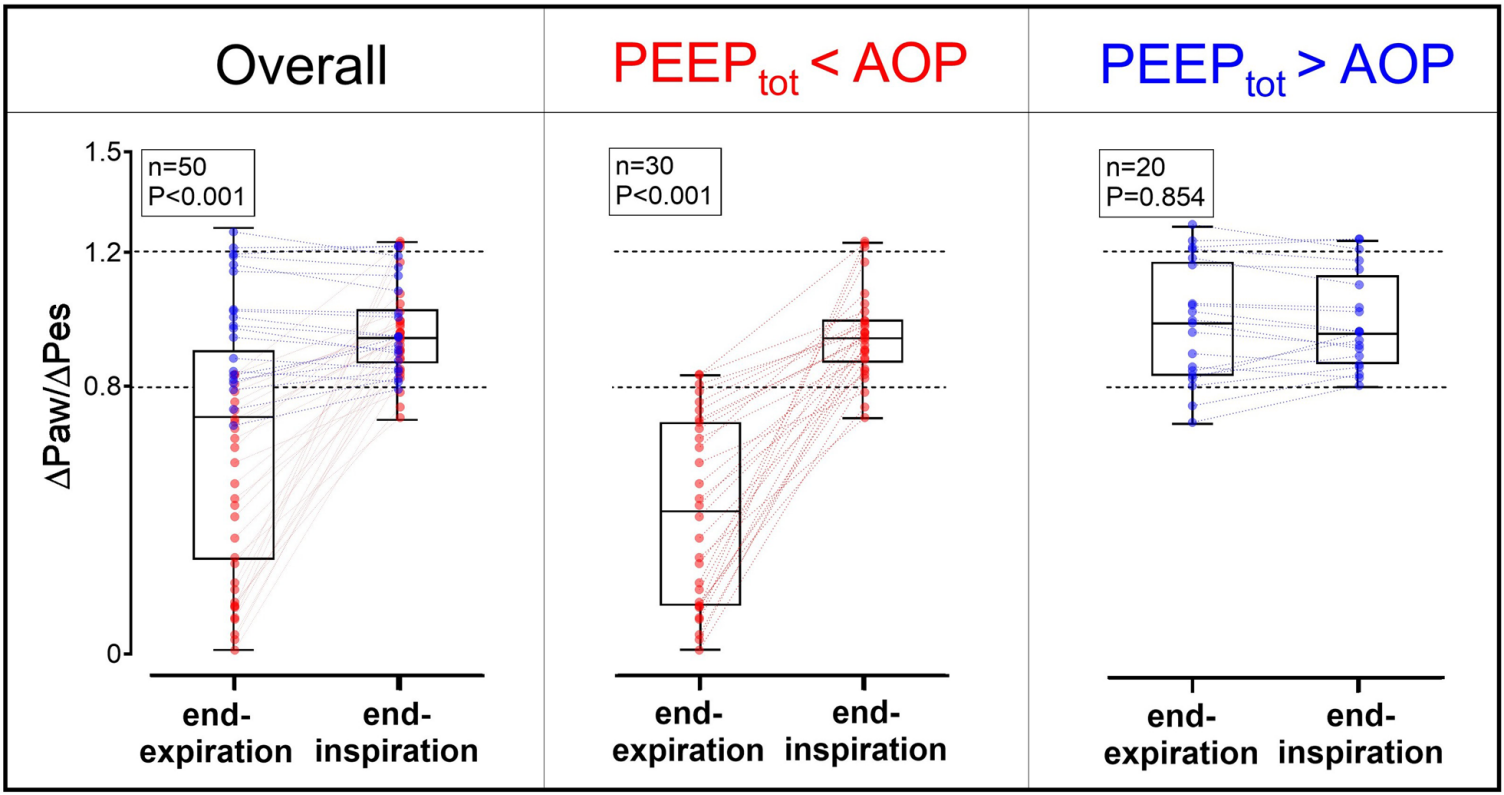
50 positive pressure occlusion tests performed by manual external compression during end-expiratory (end-exp.) versus end-inspiratory (end-insp.) occlusion at different positive end-expiratory pressure (PEEP) levels or trunk inclinations (0° vs. 30°) in 12 human cadavers. Results are presented overall (left) or sorted by total PEEP (PEEP_tot_) below or above the airway opening pressure (AOP, right). *P* value stands for linear mixed effects model significance level. ∆Paw/∆Pes, ratio of changes in airway pressure over changes in esophageal pressure during positive pressure occlusion test. The dashed lines indicate the 0.8–1.2 target range for ∆Paw/∆Pes

We analyzed 50 observations in 12 cadavers. Ventilation parameters were: total PEEP 8 (5;13) cmH_2_O, plateau pressure 21 (18;26) cmH_2_O and respiratory system compliance 33 (25;41) mL/cmH_2_O. Each cadaver displayed complete airway closure, with AOP 10 (9;12) cmH_2_O (ranging from 5 to 23 cmH_2_O). As shown in Fig. 1**,** the ∆Paw/∆Pes ratio was different when measured at end-expiration versus end-inspiration (estimated means 0.65 [0.62; 0.68] vs. 0.96 [0.93; 0.99]; *P* < 0.001). When stratifying the results according to AOP, this difference was present when total PEEP was lower than AOP (estimated means 0.42 [0.40; 0.44] vs. 0.95 [0.92; 0.97], *P* < 0.001, n = 30), and was not observed when total PEEP was higher than AOP (estimated means 0.99 [0.92; 1.05] vs. 0.99 [0.92; 1.06], *P* = 0.854, n = 20). Performing measurements at end-inspiration provided a ratio within the expected range.

### Patients

**Fig. 2:**
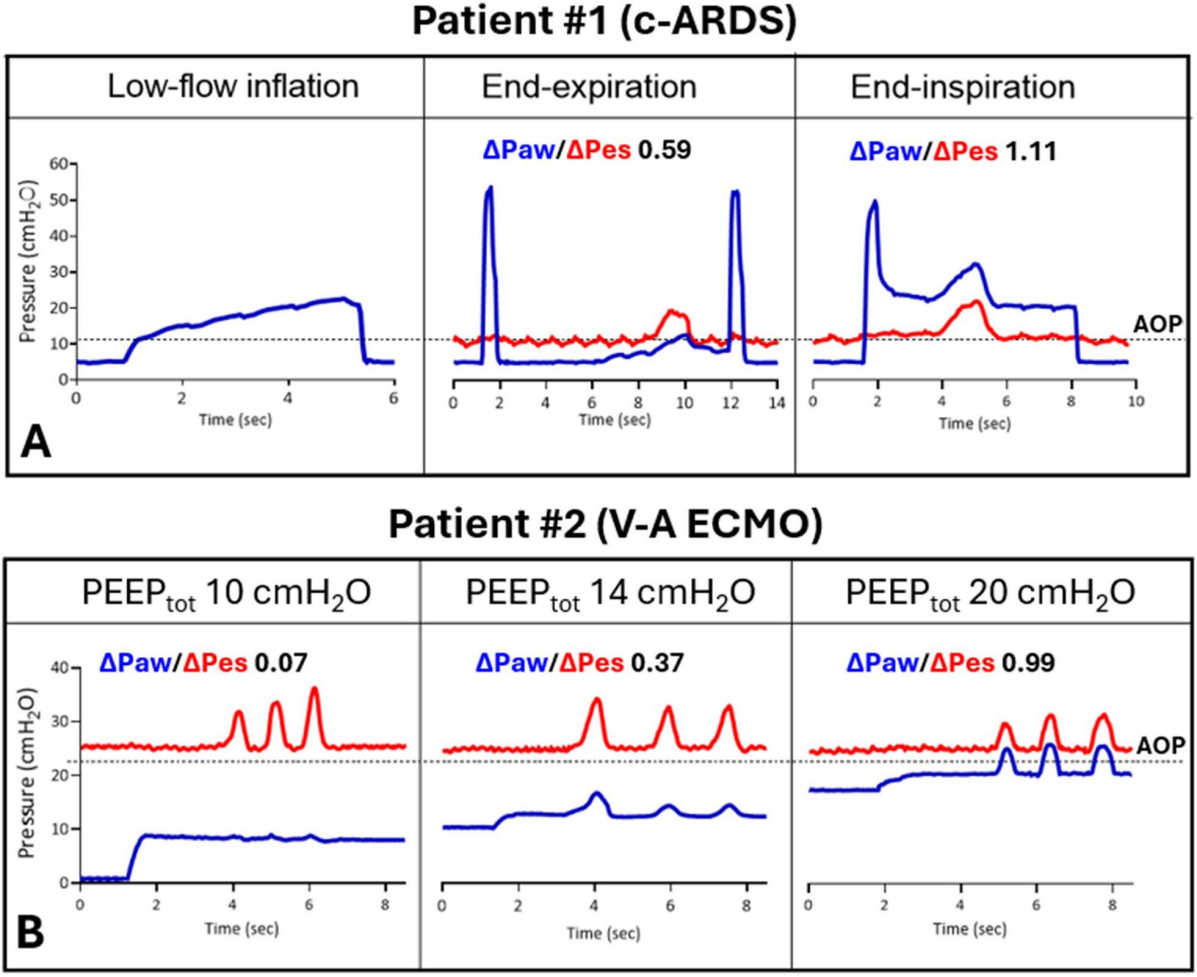
Esophageal pressure (Pes) calibration in two patients with airway closure (dashed lines). **A** In Patient #1, admitted to the Intensive Care Unit for acute respiratory distress syndrome associated to COVID-19 (c-ARDS), a low-flow inflation (left panel) revealed the presence of airway opening pressure (AOP). An esophageal balloon was correctly placed at the chest x-ray assessment. Although many attempts, it was not possible to obtain a ratio between airway and esophageal pressure swings (∆Paw/∆Pes) within 0.8 and 1.2 when performing the calibration maneuver (positive pressure occlusion test) during an end-expiratory occlusion starting from a total positive end-expiratory pressure (PEEP) of 7 cmH_2_O (middle panel). However, when the test was performed during an end-inspiratory occlusion (right panel) from a plateau pressure of 24 cmH_2_O, the calibration ratio was within the recommended values. **B** In Patient #2, requiring veno-arterial extracorporeal membrane oxygenation (V-A ECMO) after out-of-hospital cardiac arrest, AOP was found at 22 cmH_2_O. An esophageal balloon was correctly placed at the chest x-ray assessment. A positive pressure occlusion test was performed at end-expiration to verify the correct positioning of the esophageal catheter. Total PEEP (extrinsic plus intrinsic) was measured during the occlusion. At 0 cmH_2_O of extrinsic PEEP (total PEEP 10 cmH_2_O), significant swings in Pes during chest compressions were observed in absence of correspondent changes in Paw (left panel). Repeating the calibration test after extrinsic PEEP was increased to 10 cmH₂O (total PEEP 13 cmH₂O), limited swings in Paw started to appear (middle panel). After a further increase of PEEP at 17 cmH₂O (total PEEP 20 cmH₂O), a clear swing in Paw was observed and was of the same size of the Pes swing. This allowed us to achieve a reliable ∆Paw/∆Pes ratio and balloon calibration (right panel)

Figure 2 shows the waveforms recorded during Pes calibration in two passively mechanically ventilated patients with complete airway closure. In both cases, the ∆Paw/∆Pes was low and not acceptable when the occlusion test was performed below AOP. Repeating the test at a level of pressure higher than (during end-inspiratory occlusion) or comparable to AOP (at end-expiration with a higher PEEP level) allowed a reliable ∆Paw/∆Pes and ascertained balloon calibration without changing the position of the balloon.

## Discussion

The main findings of this study can be summarized as follows. When performing a positive pressure occlusion test at end-expiration to calibrate Pes during passive mechanical ventilation, the presence of airway closure limits the transmission of the chest compression to the pressure at the airway opening. This leads to a conspicuous underestimation of the ∆Paw/∆Pes if the test takes place below AOP. The clinical implication is the incorrect assumption of esophageal catheter misplacement or limited Pes interpretation.

Our findings constitute a proof-of-concept of the airway closure phenomenon: any measurement of pressure performed at the airway opening as reflecting the alveolar pressure (driving pressure, plateau pressure, total PEEP, transmission of chest compression) is correct only under the assumptions that the airways are open.

Additionally, data from the two patients highlight the clinical relevance of these findings in critical care conditions at high risk for airway closure and in which Pes monitoring may add valuable information. This is particularly relevant considering that 30–50% of ARDS patients have airway closure, especially in case of concomitant obesity (2). An unsuccessful Pes calibration test may suggest the presence of airway closure. If a formal evaluation with a low-flow inflation has not been done, performing the calibration test at end-inspiration may unveil that total PEEP is below AOP. Thus, our findings suggest that it might be more convenient to calibrate Pes by means of an end-inspiratory occlusion. Performing the calibration of the balloon at end-inspiration may be a user-friendly approach to account for airway closure even in centers in which Pes is not routinely used.

In case of severe hemodynamic instability caution should be taken not to perform a positive pressure occlusion test on top of a prolonged end-inspiratory pause, as the increase in intrathoracic pressure may not be tolerated by the patient.

The study findings are limited to passive mechanical ventilation. While Thiel cadavers are an appropriate human model of airway closure, absence of active circulation may have affected thoracic compliance minimally and does not allow to address any hemodynamic effect.

In conclusion, when performing a standard positive pressure occlusion test at end-expiration to calibrate Pes during passive mechanical ventilation, setting PEEP below AOP leads to a conspicuous underestimation of the ∆Paw/∆Pes ratio. This constitutes a proof-of-concept of the airway closure phenomenon, which may be unveiled by increasing PEEP or by performing a pressure occlusion test at end-inspiration. Our results favor either the incorporation of airway closure evaluation and a positive pressure occlusion test performed during end-inspiratory occlusion as the new standard of operative procedures for positioning and calibrating the esophageal balloon.

## Availability of data and materials:

The datasets used and/or analyzed during the current study are available from the corresponding author on reasonable request.

## Abbreviations

AOP: Airway opening pressure
ARDS: Acute respiratory distress syndrome
Paw: Airway pressure
Pes: Esophageal pressure
∆Paw/∆Pes: Esophageal pressure calibration ratio
PEEP: Positive end-expiratory pressure
